# The Treatment of Rickets

**Published:** 1892-12-17

**Authors:** 


### The Hospital Clinic.

[*The Editor will be glad to receive offers of co-operation and contributions from members of the profession. All letters should be addressed to* The Editor, The Lodge, Porchester Square, London, W.]

### HOSPITAL FOR SICK CHILDREN, GREAT ORMOND STREET.

### The Treatment of Rickets.

The treatment of rickets at Great Ormond Street Hospital for Sick Children may be conveniently summarised under the following heads: (1) The dietetic and hygienic treatment; (2) the treatment by drugs; (3) the treatment of the complications.

Success in the treatment of rickets depends mainly on a rigid and minute attention to detail in the rectification of the dietetic and hygienic conditions. The occurrence of rickets has been shown to be the result of bad feeding, both as regards quality and quantity of food; and it is, therefore, of the utmost importance that errors of diet should be corrected from the outset. At the Great Ormond Street Hospital much more stress is laid on the treatment of rickets by proper feeding than by drugs. The child may be brought for treatment under one of the following conditions: (1) It may still be suckling, or (2) it may have been weaned and then fed by hand; (3) it may have been hand-fed from its birth; (4) it may suffer from chronic diarrhœa and vomiting; (5) it may be suffering from congenital syphilis.

If the child is still suckling, either the mother, owing to ill-health or to the fact that she is again pregnant, is unable to provide sufficient good milk; or the child has been suckling too long.

In the first case, additional milk must, of course, be given to the child, prepared in the manner described below. In the second case, the child must be weaned, and food of proper quality supplied to it. As a rule, a child should be weaned when ten months old. In the second and third of the above-mentioned conditions the rickets has been produced by unsuitable feeding. If the child is being brought up by hand it should be fed on cow's milk, warmed and diluted, the amount of dilution being regulated by the age of the child. Fresh cow's milk must always be employed, and must be boiled before being used. During the first month about a pint of milk may be given during the 24 hours, the child being fed every two hours. The milk may be diluted with water alone, or with lime or barley water, the two latter being the more suitable. The proportion of milk to lime water during the first month must be as one to two. The amount of milk given and the proportion of milk to lime water should be gradually increased during each successive month. After the first two months the child need not be fed more often than every three hours. The addition of a little sugar to each bottle is required, and the nutritive value of milk thus prepared is increased by a tablespoonful of cream. In those cases in which cow's milk cannot be taken, condensed milk diluted in the proportion of one of milk to fifteen of water may be tried temporarily. Feeding with cow's milk should be again attempted after a few weeks' interval.

The majority of the cases of rickets come under observation at the age of eleven months and upwards. The cause is generally to be traced to improper and unsuitable food. At this age the child is able to digest good milk, and this should if possible be prescribed. In a large number of the cases treated at Great Ormond Street the child is unable to digest a sufficient quantity of milk to supply the requisite amount of fat and nitrogenous material required. Under these circumstances the deficiency in these articles of food must be supplied by other means. Cream may be given to make up for the deficiency in fat, and raw meat pulp, or raw meat juice is prescribed in order to supply a sufficient quantity of nitrogenous material. Raw meat pulp is made at Great Ormond Street as follows: The raw muscle is scraped, and by this means the soft muscle elements are obtained, free from fibre and tendon. This is best given in a raw state; two ounces of the pulp is an average daily quantity for a child of twelve months.

The beef juice may be obtained by scraping two ounces of meat as above, and adding half an ounce of cold water. Let this stand one hour, and then squeeze the whole through fine muslin. Two to three ounces of the juice are given in the twenty-four hours. It is most essential that it is freshly prepared. The taste is disguised by giving it with milk, or by sweetening it with a little malt extract. The earthy salts required are contained in sufficient quantity in the milk, cream, and meat juice thus administered.

If the child comes for treatment with diarrhœa and vomiting, it is put on a suitable quantity of milk, properly diluted, and the symptoms further treated by medicinal means. If syphilis be the cause of the malnutrition, it is treated with ordinary anti-syphilitic remedies.

The most perfect hygienic conditions possible and the most scrupulous cleanliness, both as regards the child, its food, and the feeding utensils, are insisted on. A tepid bath is ordered to be given at least once a day, and the body of the child protected by flannel worn next the skin. Exercise is most beneficial, and the child should have plenty of fresh air and sunlight.

